# The revised Green *et al.*, Paranoid Thoughts Scale (R-GPTS): psychometric properties, severity ranges, and clinical cut-offs

**DOI:** 10.1017/S0033291719003155

**Published:** 2021-01

**Authors:** Daniel Freeman, Bao S. Loe, David Kingdon, Helen Startup, Andrew Molodynski, Laina Rosebrock, Poppy Brown, Bryony Sheaves, Felicity Waite, Jessica C. Bird

**Affiliations:** 1 Department of Psychiatry, University of Oxford; 2 Oxford Health NHS Foundation Trust, Oxford, UK; 3 The Psychometrics Centre, University of Cambridge, Cambridge, UK; 4 Academic Department of Psychiatry, Faculty of Medicine, University of Southampton, Southampton, UK; 5 Sussex Partnership NHS Trust, UK

**Keywords:** Assessment, delusions, paranoia

## Abstract

**Background:**

The Green *et al*., Paranoid Thoughts Scale (GPTS) – comprising two 16-item scales assessing ideas of reference (Part A) and ideas of persecution (Part B) – was developed over a decade ago. Our aim was to conduct the first large-scale psychometric evaluation.

**Methods:**

In total, 10 551 individuals provided GPTS data. Four hundred and twenty-two patients with psychosis and 805 non-clinical individuals completed GPTS Parts A and B. An additional 1743 patients with psychosis and 7581 non-clinical individuals completed GPTS Part B. Factor analysis, item response theory, and receiver operating characteristic analyses were conducted.

**Results:**

The original two-factor structure of the GPTS had an inadequate model fit: Part A did not form a unidimensional scale and multiple items were locally dependant. A Revised-GPTS (R-GPTS) was formed, comprising eight-item ideas of reference and 10-item ideas of persecution subscales, which had an excellent model fit. All items in the new Reference (*a* = 2.09–3.67) and Persecution (*a* = 2.37–4.38) scales were strongly discriminative of shifts in paranoia and had high reliability across the spectrum of severity (*a* > 0.90). The R-GPTS score ranges are: average (Reference: 0–9; Persecution: 0–4); elevated (Reference: 10–15; Persecution: 5–10); moderately severe (Reference: 16–20; Persecution:11–17); severe (Reference: 21–24; Persecution: 18–27); and very severe (Reference: 25+; Persecution: 28+). Recommended cut-offs on the persecution scale are 11 to discriminate clinical levels of persecutory ideation and 18 for a likely persecutory delusion.

**Conclusions:**

The psychometric evaluation indicated a need to improve the GPTS. The R-GPTS is a more precise measure, has excellent psychometric properties, and is recommended for future studies of paranoia.

## Introduction

Trust connects individuals, but the obverse – mistrust – disconnects. Our view is that excessive mistrust, paranoia, is corrosive for mental health, relationships, and societal well-being. Many people have a few paranoid thoughts, a few people have many (Freeman *et al*., 2005). Excessive mistrust is common in adolescents (Wong *et al*., 2014; Bird *et al*., 2019), adults (Bebbington *et al*., 2013; Elahi *et al*., 2017), and older adults (Östling and Skoog, 2002; Cohen *et al*., 2004). Paranoia in its severest form, persecutory delusion, is seen clinically in conditions such as schizophrenia. Over the past 20 years, paranoia has become an increasing focus of research. As part of this research endeavor, the Green *et al*., Paranoid Thoughts Scale (GPTS; Green *et al*., 2008) was developed to assess paranoia across the spectrum of severity. Published in this journal, the GPTS is recommended as the best current measure of paranoia (Statham *et al*., 2019). In this paper, we use data collected over the past decade to rigorously assess the psychometric properties of the GPTS and provide score ranges with clinical cut-offs to enable interpretation of scale scores.

The central focus of the GPTS is on the occurrence of recent persecutory ideation (Part B), since this is the content of persecutory delusions. Scale items were generated based on a definition that persecutory ideation consists of believing that harm is going to occur and that the perpetrator has the deliberate intention to cause this harm (Freeman and Garety, 2000). It is the strength of the GPTS Part B questionnaire that all item content is clearly of a persecutory nature (e.g. ‘Certain individuals have had it in for me.’ ‘People wanted me to feel threatened, so they stared at me.’), whereas older scales such as the Paranoia Scale (Fenigstein and Vanable, 1992) predominately contain items that do not meet the definition of persecutory ideation (e.g. ‘No one really cares much what happens to you’, ‘I am sure I get a raw deal from life’). The development of the GPTS was also influenced by a theoretical perspective that there is a hierarchy of paranoia (Freeman *et al*., 2005): persecutory ideation typically builds upon ideas of reference and other social-evaluative concerns. The results of factor analytic studies support this coupling of ideas of reference and persecution (e.g. Paolini *et al*., 2016). Hence the GPTS also included a scale assessing the related but less severe phenomenon of social ideas of reference (Part A). Item content for Part A was developed in line with criteria building on the work of Startup and Startup (2005): ‘The person holds the belief that some neutral event has special personal significance/refers to them personally by means of observation or communication by another.’ The items were also designed to capture the dimensions of conviction, preoccupation, and distress. In the initial validation, principal components analysis of a 93-item pool for the GPTS completed by 353 university students indicated two components: persecution and social ideas of reference. The item pool was also completed by 50 patients with current persecutory delusions in the context of psychosis (Green *et al*., 2008). Items for the two 16-item Part A (reference) and Part B (persecution) scales were selected by considering: factor loadings, item-scale correlation, item variance, level of item endorsement, ability to discriminate between the non-clinical and clinical group, and overall face validity of the scales. In the original scale paper, the GPTS demonstrated good psychometric properties, including test-retest reliability, convergent validity, and sensitivity to change. Further confirming the construct validity of the scale, GPTS scores are associated with the occurrence of unfounded paranoia in virtual reality simulations of neutral social situations (e.g. Freeman *et al*., 2008; Freeman *et al*., 2010).

The GPTS is now the most commonly used measure of paranoia in research studies (e.g. Scott *et al*., 2017; Raihani and Bell, 2018; Rehman *et al*., 2018) and clinical trials (e.g. Freeman *et al*., 2015*a*, 2015*b*, 2015*c*; van den Berg *et al*., 2016; Garety *et al*., 2017). In a recent review of self-report measures of paranoia, Statham *et al*. (2019) conclude: ‘on the basis of current evidence, the GPTS offers the most valid and informative assessment of paranoia. However some psychometric findings (e.g. internal consistency, structural validity) require replication with a larger sample.’ In this paper, we pool our data over the past decade from both clinical studies of psychosis and of non-clinical paranoia in the general population to evaluate the psychometric properties of the GPTS. We had three objectives. First, we wanted to assess the validity of the basic two-factor structure of the GPTS with a much larger sample size. Second, we wanted to use item response theory (IRT) (Reise and Henson, 2003) to evaluate item properties, test reliability, and measurement invariance between different ages and genders. Third, we wanted to enable the interpretation of scale scores by specifying severity ranges. In future research, it would be valuable to know the level of the paranoia spectrum that is being studied, since this is opaque in many non-clinical studies that either do not select for paranoia score or use median splits to categorize purportedly ‘high’ and ‘low’ paranoia groups. In clinical studies and settings, it would also be valuable to have a screening tool for whether an individual is likely to have a persecutory delusion.

## Method

### Participants

There were a total of 10 551 participants with GPTS data from 16 studies. In total, 1228 participants completed both GPTS Part A (ideas of reference) and Part B (ideas of persecution). Of these participants, 1212 had complete data (with no missing items) for both Parts A and B, 1218 had complete data (with no missing items) for Part A, and 1221 had complete data (with no missing items) for Part B. This included 806 participants recruited from the general population from four studies (Freeman *et al*., 2008, 2013*b*, 2015*b*; and one yet unpublished) and 422 patients with psychosis from eight studies (Freeman *et al*., 2014, 2015*a*, 2015*c*, 2016, 2016*a*, 2016*b*, 2019*a*; Bradley *et al*., 2018). An additional 9324 participants provided complete GPTS data with no missing items for Part B only, including 3826 individuals from two general population studies (Freeman *et al*., 2013*a*; Brown *et al*., submitted), 3755 university students with insomnia (Freeman *et al*., 2017), and 1743 patients with non-affective psychosis (Freeman *et al*., 2019*b*). This provided a total of 10 545 participants with complete data for the GPTS Part B.

#### Subgroups

Participant subgroups, based on clinical information, were created for descriptive reports and the ROC analysis. Nine hundred and thirty-seven participants from three general population studies who reported non-psychotic mental health disorders were included in a mental health problems subgroup. This included 32 people who reported being treated for anxiety and/or depression (Freeman *et al*., 2013*b*), 236 participants who reported current (non-psychotic) mental health problems (Freeman *et al*., 2013*a*), and 669 participants reporting current contact with mental health services for (non-psychotic) mental health problems (Freeman *et al*., 2017). Participants who reported not having any mental health problems and participants from the remaining general population studies with no mental health information formed a non-clinical subgroup (*n* = 7297). The psychosis subgroup consisted of 1804 patients from three clinical studies who had been recruited based on a diagnosis of psychotic disorder (Freeman *et al*., 2015*c*, 2019*a*; Bradley *et al*., 2018). The persecutory delusion group consisted of 360 patients with psychosis, from six clinical trials, recruited for the presence of a persecutory delusion (Freeman *et al*., 2014, 2015*a*, 2016, 2016*a*, 2016*b*, 2019*a*). One hundred and forty-seven participants from the general population studies were not included in any of the subgroups due to a self-reported personal or family history of psychosis. This included 45 participants with a diagnosis of a severe mental illness such as bipolar disorder or schizophrenia (Brown *et al*., submitted), nine participants with a diagnosis of a psychotic disorder (Freeman *et al*., 2017), and 93 participants with a reported family history of psychosis (Freeman *et al*., 2013*a*).

### Measure

#### Green *et al*. Paranoid Thoughts Scale

The GPTS is a thirty-two item self-report measure of paranoia, designed for both clinical and non-clinical populations (Green *et al*., 2008). Part A assesses ideas of reference (e.g. ‘It was hard to stop thinking about people talking about me behind my back’) and Part B assesses ideas of persecution (e.g. ‘I was convinced there was a conspiracy against me’). Each item is rated on a five-point scale (1–5). Scores on each scale can range from 16 to 80. Higher scores indicate greater levels of paranoid thinking.

### Analysis

All analyses were conducted in R, version 3.5 (R Core Team, 2013). Packages used included ‘psych’ (Revelle, 2018), ‘mirt’ (Chalmers, 2012), ‘pROC’ (Robin *et al*., 2011), and ‘optimumCutpoints’ (Lopez-Raton *et al*., 2014).

#### Factor structure

The factor structure of the GPTS was assessed in the 1212 participants with complete GPTS data from both Parts A and B. Factor analysis was appropriate as Bartlett's test of Sphericity was significant (χ^*2*^ = 46 249.4, df = 496, *p* < 0.001) and the Kaiser–Meyer–Olkin test of sampling adequacy was excellent (KMO = 0.98). Confirmatory factor analysis (CFA) using the MLR robust maximum likelihood estimator was first conducted to examine the model fit of the two-factor structure identified in the initial GPTS validation study (Green *et al*., 2008). Model fit was assessed using a Comparative Fit Index (CFI) and Tucker–Lewis index (TLI) of >0.95, a Root Mean Square Error of Approximation (RMSEA) of <0.06, and a Standardized Root Mean Square Residual (SRMR) of <0.08 (Hu and Bentler, 1999). Based on the outcome of the CFA, exploratory factor analysis (EFA) was then conducted using principal axis factoring and oblique rotation. For the revised GPTS, items were considered for deletion by assessing the factor loadings, residuals, and content of items.

#### IRT analysis

There are a number of helpful introductory and detailed descriptions of IRT techniques available (e.g. Reise and Waller, 2009; Embretson and Reise, 2013; van der Linden and Hambleton, 2013). IRT analyses were conducted using all available data for each subscale of the GPTS (Part A = 1218, Part B = 10 545). Where appropriate, unidimensional IRT analyses were conducted to examine the item and test properties of the individual factors of the GPTS. IRT was only conducted if the assumption of unidimensionality was met. The EFA and Mokken analysis were used to evaluate whether items conform to a single scale, with Loevinger's H above 0.3 indicating unidimensionality (Stochl *et al*., 2012). A two-parameter graded response model (GRM) was fitted to the items (Samejima, 1969). Person fit statistics were calculated to detect outliers where the pattern of responses across the items was atypical and therefore likely guided by other response mechanisms (e.g. random responding). Participants with atypical response patterns, determined by extreme person fit statistic scores (*z* < −3 or >3), were excluded (Felt *et al*., 2017).

The item and test parameters derived from the IRT analysis are expressed as a function of *θ*, representing the continuum of the latent trait (i.e. paranoia) where values denote standard deviations from the average level (*θ* = 0). As such, higher values of *θ* represent more severe paranoia. The ability of each item to discriminate different levels of paranoia is denoted by the discrimination parameter (*a*), with higher values indicating small shifts in severity lead to increases in the probability that an item will be endorsed. Discrimination parameters above 1 are highly discriminative, whilst those below 0.5 are considered unacceptable (Baker and Kim, 2017). The difficulty parameters (*b*) describe the level of severity that the item measures, with the four difficulty parameters for each item denoting the 50% probability of responding at the boundary between each response option. Higher difficulty parameters indicate that the item responses typically measure more severe levels of paranoia.

The reliability of the GPTS was evaluated using the test information (TI) function, representing the precision of the measure at different points along the *θ* spectrum. To aid interpretation, the TI at specific values of *θ* were converted to an equivalent *α* reliability on a 0–1 scale with the formula 1/√TI(θ) (O'Connor, 2018). To evaluate measurement invariance, we conducted differential item functioning (DIF) analysis for age and gender, with the criteria of a *β* change above 10% and a pseudo *R*^2^ above 0.13 indicating significant item variance (Crane *et al*., 2007; Choi *et al*., 2011). The presence of DIF reflects a measurement bias where demographic factors influence the way participants respond to the items (Holland and Wainer, 2012).

#### Determining score ranges and clinical cut-offs

The expected score function from the IRT analysis was used to examine score ranges, providing the expected total score at different points of the *θ* spectrum. To assess the accuracy of the expected score function, we examined the model fit of the GRM for the data and the correlation between *θ* scores and raw total scores. Receiver operating characteristic (ROC) analyses were conducted using the 360 patients with a confirmed persecutory delusion as the discrimination group and non-clinical participants from the general population (*n* = 7297) as the control group. The area under the curve (AUC) was used to evaluate the ability of the GPTS to discriminate people with persecutory delusions from the control group, with values above 0.70 considered fair, over 0.80 good, and over 0.90 excellent (Egan, 1975). The cut-off score providing the optimal balance of sensitivity and specificity was then calculated based on Youden's *J* statistic (Youden, 1950). Cut-off scores were incorporated with the expected score function to determine the score ranges.

## Results

### The GPTS

#### Factor structure

The initial CFA in the 1212 participants with complete data for the full GPTS demonstrated the original two-factor structure of Part A and B had an inadequate model fit (χ^2^ = 2599.2, df = 463, CFI = 0.91, TLI = 0.90, RMSEA = 0.087, SRMR = 0.038). An EFA was therefore conducted on the 32 items to explore the factor structure (see online Supplementary Materials). Although a parallel analysis suggested a four-factor model, none of the 32 items loaded uniquely on a third or fourth factor when these solutions were extracted. As a result, a two-factor model was still considered the best solution. This identified that although all 16 persecution items strongly loaded on the same factor with no cross-loadings, only 10 of the social reference items loaded onto a unique factor. Within the social reference scale, four items loaded on both factors (‘*I was convinced that people were singling me out*’, ‘*People have been checking up on me*’, ‘*I was stressed by people watching me*’, and ‘*I was worried by people*'*s undue interest in me*’), and two items loaded only on the persecution factor (‘*I was certain that people have followed me*’ and ‘*Certain people were hostile towards me personally*’).

These findings suggest that the 16 social reference items do not have a coherent factor structure and therefore cannot be considered a unidimensional scale. However, as all 16 persecution items loaded strongly on one factor that can be treated as a unidimensional subscale to measure ideas of persecution. Mokken analysis confirmed all 16 persecution items were within a single factor, with Loevinger's *H* coefficients above 0.3 for all items and an overall coefficient of homogeneity of 0.699 (s.e. = 0.005). Mean GPTS persecution scores for each of the four participant subgroups are shown in Table 1.

**Table 1.** Mean scores for the original GPTS and Revised-GPTS for participant subgroups

Group	GPTS Persecution (16–80)		R-GPTS Social reference (0–32)		R-GPTS Persecution (0–40)	
	Mean (s.d.)	*n*	Mean (s.d.)	*n*	Mean (s.d.)	*n*
General population	22.8 (10.6)	7297	6.77 (5.54)	774	4.52 (6.74)	7297
Mental health problems	28.7 (14.9)	982	9.43 (8.06)	32	8.21 (9.35)	982
Psychosis	38.1 (21.1)	1804	15.8 (7.42)	62	13.7 (13.0)	1804
Persecutory delusions	58.7 (14.8)	360	19.9 (7.80)	360	26.1 (9.46)	360

#### Psychometric properties

We report the properties of the GPTS Persecution scale (Part B), which can inform the understanding of previous studies that have used this scale. Although the 16 items had a coherent unidimensional factor structure, several items had correlated residuals (Yen's Q3>0.2), suggesting local dependence within the items. The IRT analysis should therefore be interpreted with caution. Following the removal of participants with atypical response patterns (*n* = 190), a GRM with the remaining 10 355 participants demonstrated a good fit to the data (CFI = 0.99, TLI = 0.98, SRMSR = 0.037, RMSEA = 0.068).

The item parameters for the GPTS Persecution scale are provided in the online Supplementary Materials. All 16 items were highly discriminative of shifts in paranoia (*a* = 2.45–5.37). The most discriminating items were ‘*I was distressed by being persecuted*’ (*a* = 5.37, s.e. = 0.12) and ‘*The thought that people were persecuting me played on my mind*’ (*a* = 5.07, s.e. = 0.11). High difficulty parameters for a response of 0–1 (*b*^1^) on the items ‘*I was convinced there was a conspiracy against me*’ (*b*^1^ = 0.79, s.e. = 0.02), ‘*I was sure someone wanted to hurt me*’ (*b*^1^ = 0.78, s.e. = 0.01), ‘*People wanted me to feel threatened, so they stared at me*’ (*b*^1^ = 0.76, s.e. = 0.02), and ‘*I was distressed by being persecuted*’ (*b*^1^ = 0.76, s.e. = 0.01) suggest any endorsement of these items, even at a low level, are indicative of high paranoia severity (>0.75 s.d. above average). In contrast, low-level endorsement on the items ‘*I was certain people did things in order to annoy me*’ (*b*^1^ = −0.09, s.e. = 0.02) and ‘*Certain individuals have had it in for me*’ (*b*^1^ = 0.22, s.e. = 0.01) is in line with average levels of paranoia in the population. For each of the 16 items, full endorsement (*b*^4^; response of 4) indicates a severe level of persecutory ideation (>1.50 s.d. above average).

Overall reliability was high across the spectrum of paranoia severity, with *α* values >0.90 within the *θ* range of 0.27 below and 2.51 s.d. above average levels of paranoia, and *α* >0.95 between 0.015 below and 2.29 s.d. above average. This shows the persecution scale is most reliable at heightened levels of severity, with a maximum *α* of 0.99 (TI = 78.3, s.e. = 0.11) at 1.14 s.d. above average paranoia. All 16 persecution items were invariant between men (*n* = 3677) and women (*n* = 4830), and between age groups (13–21 years, *n* = 2636; 22–29 years, *n* = 3047; 30–44 years, *n* = 2440; 45+ years, *n* = 2232), in the DIF analysis (pseudo *R*^2^ change <0.13 and *β* change <10%).

#### Score ranges

The total score from the 16 original persecution items was highly correlated with the participant *θ* scores (*r* = 0.90), indicating the total score has a high level of precision. The expected score function (supplementary materials) showed most people are unlikely to endorse many persecution items, with an expected score of 19.1 (minimum 16) at the average level of paranoia in the population. Expected scores increase as the level of trait paranoia increases, with expected scores of 26.7 at 0.5 s.d. above average, 42.6 at 1.0 s.d. above average, 60.9 at 1.5 s.d. above average, and 74.1 at 2.0 s.d. above average.

The GPTS Persecution score ranges are shown in Table 2. Our recommended cut-off for identifying moderately severe persecutory ideation is a score of 35 or above, representing 0.80 s.d. above the average level of paranoia in the population. ROC analysis identified 35 as the optimal cut-off point (sensitivity = 0.931, 95% CI 0.903–0.955; specificity = 0.878, 95% CI 0.870–0.885) to discriminate patients with persecutory delusions (*n* = 360) from the non-clinical group (*n* = 7297), with an overall AUC of 0.959 (95% CI 0.950–0.969).

**Table 2.** Suggested score categories for the original GPTS Persecution scale (16 items) and the Revised GPTS Persecution (10 items) and Reference (eight items) scales

Category	*θ* range	GPTS Persecution (16–80)	R-GPTS Persecution (0–40)	R-GPTS Reference (0–32)
Average	<0.35	16–23	0–5	0–9
Elevated	0.40–0.75	24–34	6–10	10–15
Moderately severe	0.80–1.05	35–44	11–17	16–20
Severe	1.10–1.45	45–59	18–27	21–24
Very severe	>1.50	60+	28+	25+
Cut-off for persecutory delusions		45	18	

*Note*. The *θ* values represent standard deviations above the average (*θ* = 0) population level.

Although a score of 35 most accurately discriminates the delusion group from a non-clinical sample, individuals with persecutory delusions typically score well above this level with a mean score of 58.7 (s.d. = 14.8) and a lower quartile of 49. Our recommended cut-off to identify severe persecutory ideation and the likely presence of a persecutory delusion is 45, representing 1.10 s.d. above the average level of paranoia in the population. The ROC analysis demonstrates that a cut-off of 45 is unlikely to incorrectly identify an individual as having a persecutory delusion when they do not (specificity = 0.94, 95% CI 0.93–0.95), while still being able to identify the majority of patients with confirmed persecutory delusions (sensitivity = 0.81, 95% CI 0.77–0.85). As shown in Table 3, scores above this level were present in 81% (*n* = 293) of the patients with persecutory delusions.

**Table 3.** Proportions of participants scoring above the thresholds for each score range for the GPTS persecution scale and R-GPTS persecution and reference scales

	Non-clinical		Mental health problems		Psychosis		Persecutory delusions	
	*n*	%	*n*	%	*n*	%	*n*	%
GPTS Persecution								
⩽23 (within average range)	5255	72%	515	52%	671	37%	4	1%
24+ (elevated+)	2042	28%	467	48%	1133	63%	356	99%
35+ (moderately severe+)	891	12%	258	26%	841	47%	335	93%
45+ (severe+)	439	6%	151	15%	662	37%	293	81%
60+ (very severe+)	107	1%	58	6%	366	20%	197	55%
R-GPTS Persecution								
⩽5 (within average range)	5296	73%	529	54%	715	40%	8	2%
6+ (elevated+)	2001	27%	453	46%	1089	60%	352	98%
11+ (moderately severe+)	1077	15%	298	30%	883	49%	334	93%
18+ (severe+)	484	7%	165	17%	650	36%	292	81%
28+ (very severe+)	106	1%	61	6%	352	20%	182	51%
R-GPTS Reference								
⩽9 (within average range)	590	77%	19	59%	13	21%	39	11%
10+ (elevated+)	181	23%	13	41%	49	79%	320	89%
16+ (moderately severe+)	59	8%	5	16%	31	50%	248	69%
21+ (severe+)	25	3%	5	16%	16	26%	179	50%
25+ (very severe+)	15	2%	2	6%	10	16%	109	30%

### The Revised GPTS

Due to the problematic factor structure of the GPTS Part A and the local dependence in items in Part B, we derived a Revised-Green *et al*., Paranoid Thoughts Scale (R-GPTS). The six items from Part A that loaded on the persecution factor in the initial EFA were deleted, providing a clean two-factor structure. Five Part B items were deleted due to highly correlated residuals with other items (‘*I have definitely been persecuted*’, ‘*People have intended me harm*’, ‘*I was distressed by people wanting to harm me in some way*’, ‘*I was annoyed because others wanted to deliberately upset me*’, ‘*The thought that people were persecuting me played on my mind*’). One further item was deleted from Part B due to potentially confusing wording (‘*I was preoccupied with thoughts of people trying to upset me deliberately*’). Two additional social reference items were deleted due to loading on the persecution factor in the revised EFA (‘*I was frustrated by people laughing at me*’ and ‘*It was hard to stop thinking about people talking about me behind my back*’).

Parallel analysis of the remaining 18 items suggested a two-factor model was now the best solution (see online Supplementary Materials). The final model with an eight-item Reference scale and a 10-item Persecution scale provided a clean factor structure explaining 69% of the variance with a good model fit (χ^2^ = 535.3, df = 134, *p* < 0.001, CFI = 0.96, TLI = 0.96, RMSEA = 0.068, SRMR = 0.031). None of the items in either revised subscale had correlated residuals above 0.20. Mokken analysis confirmed the subscales can be treated as unidimensional constructs, with Loevinger's *H* coefficients above 0.3 for all items and high overall coefficients of homogeneity (Reference: *H* = 0.637, s.e. = 0.013; Persecution: *H* = 0.675, s.e. = 0.005). The two factors were highly correlated (*r* = 0.79). The scaling of the individual items was changed to 0–4 to enable easier interpretation of total scores.

### R-GPTS reference scale

#### Psychometric properties

IRT was conducted with the 1224 participants with complete data for the eight R-GPTS Reference items. Following removal of participants with atypical response patterns (*n* = 4), a GRM demonstrated a good model fit to the items (CFI = 0.99, TLI = 0.99, SRMSR = 0.028, RMSEA = 0.064).

**Fig. 1.**
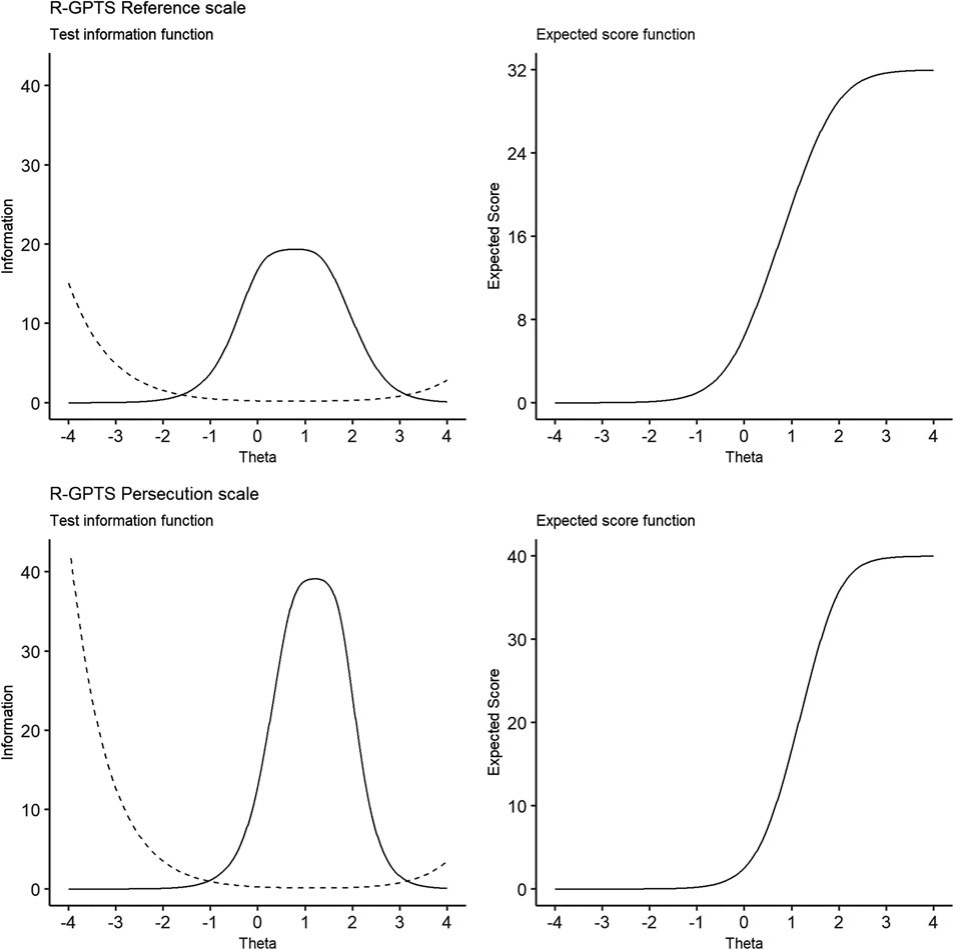
Test information (TI) with standard errors (----) and expected score across the *θ* distribution for the original GPTS Persecution scale and the Revised GPTS Reference and Persecution scales.

All eight items were highly discriminative of ideas of social reference, with parameters ranging from 2.10 to 3.69 (Table 4). The most highly discriminating item was ‘*People definitely laughed at me behind my back*’ (*a* = 3.69, s.e. = 0.21). Unlike the persecution scale, the difficulty parameters show for all eight items, low-level endorsement (response 0–1) likely represents average levels of ideas of reference within the population (*b*^1^ = −0.50 to 0.30). The items where moderate endorsement (*b*^2^ and *b*^3^) most strongly represents heightened severity were ‘*People have been dropping hints for me*’ (*b*^2^ = 0.83, s.e. = 0.05; *b*^3^ = 1.31, s.e. = 0.06) and ‘*I have been thinking a lot about people avoiding me*’ (*b*^2^ = 0.66, s.e. = 0.05; *b*^3^ = 1.26, s.e. = 0.06). For each of the eight items, full endorsement (response 3–4) represents more severe ideas of reference (>1.20 s.d. above average). The R-GPTS Reference scale has good reliability across a wide range of the spectrum of ideas of reference, with *α* values above 0.90 within 0.47 s.d. below and 2.03 s.d. above average levels of social reference (Fig. 1). The maximum reliability was 0.95 (TI = 19.4, s.e. = 0.23) at 0.82 s.d. above average.

**Table 4.** Item properties for the R-GPTS

R-GPTS	*a*	*b*1	*b*2	*b*3	*b*4
*Reference*					
1. I spent time thinking about friends gossiping about me	2.65 (0.14)	−0.10 (0.04)	0.44 (0.04)	1.17 (0.06)	1.69 (0.07)
2. I often heard people referring to me.	2.55 (0.13)	−0.12 (0.04)	0.53 (0.05)	1.13 (0.06)	1.74 (0.08)
3. I have been upset by friends and colleagues judging me critically.	2.40 (0.13)	−0.17 (0.04)	0.48 (0.05)	1.09 (0.06)	1.67(0.08)
4. People definitely laughed at me behind my back.	3.69 (0.21)	0.17 (0.04)	0.62 (0.04)	0.97 (0.05)	1.38 (0.06)
5. I have been thinking a lot about people avoiding me.	2.47 (0.13)	0.10 (0.04)	0.66 (0.05)	1.26 (0.06)	1.84 (0.08)
6. People have been dropping hints for me	2.80 (0.16)	0.31 (0.04)	0.83 (0.05)	1.31 (0.06)	1.84 (0.08)
7. I believed that certain people were not what they seemed.	2.10 (0.11)	−0.51 (0.05)	0.10 (0.05)	0.73 (0.05)	1.32 (0.07)
8. People talking about me behind my back upset me	3.37 (0.18)	−0.07 (0.04)	0.36 (0.04)	0.80 (0.05)	1.22 (0.06)
*Persecution*					
1. Certain individuals have had it in for me	2.95 (0.06)	0.20 (0.01)	0.71 (0.02)	1.28 (0.02)	1.70 (0.03)
2. People wanted me to feel threatened, so they stared at me.	2.87 (0.06)	0.75 (0.02)	1.14 (0.02)	1.58 (0.02)	2.02 (0.03)
3. I was certain people did things in order to annoy me	2.43 (0.04)	−0.10 (0.02)	0.46 (0.02)	1.08 (0.02)	1.61 (0.03)
4. I was convinced there was a conspiracy against me.	3.68 (0.08)	0.79 (0.02)	1.08 (0.02)	1.43 (0.02)	1.75 (0.03)
5. I was sure someone wanted to hurt me	4.25 (0.10)	0.78 (0.01)	1.08 (0.02)	1.39 (0.02)	1.68 (0.03)
6. I couldn't stop thinking about people wanting to confuse me	3.15 (0.07)	0.65 (0.02)	1.02 (0.02)	1.49 (0.02)	1.89 (0.03)
7. I was distressed by being persecuted	4.45 (0.10)	0.77 (0.01)	1.07 (0.02)	1.40 (0.02)	1.75 (0.02)
8. It was difficult to stop thinking about people wanting to make me feel bad	3.99 (0.08)	0.40 (0.01)	0.79 (0.02)	1.21 (0.02)	1.59 (0.02)
9. People have been hostile towards me on purpose.	3.54 (0.07)	0.35 (0.01)	0.79 (0.02)	1.24 (0.02)	1.63 (0.02)
10. I was angry that someone wanted to hurt me.	3.59 (0.08)	0.63 (0.01)	0.98 (0.02)	1.35 (0.02)	1.69 (0.02)

*Note*: *a* = discrimination, *b* = difficulty parameters at the category thresholds between 0–1 (*b*_1_), 1–2 (*b*_2_), 2–3 (*b*_3_), and 3–4 (*b*_4_).

All R-GPTS Reference items were invariant between men (*n* = 644) and women (*n* = 584), and between age groups (15–28 years, *n* = 309; 29–39 years, *n* = 315; 40–50 years, *n* = 303; 51+ years, *n* = 301), in the DIF analysis (pseudo *R*^2^ change <0.13 and *β* change <10%).

### R-GPTS persecution scale

#### Psychometric properties

IRT analysis was conducted with the 10 revised persecution items. Following removal of participants with atypical response patterns (*n* = 54), a GRM with the remaining 10 491 participants demonstrated a good fit to the data (CFI = 0.99, TLI = 0.99, SRMSR = 0.030, RMSEA = 0.062).

The item parameters are shown in Table 4. All 10 persecution items were highly discriminative of shifts in paranoia, with parameters ranging from (*a* = 2.43–4.45). As with the original GPTS Persecution scale, the most discriminating item was still ‘*I was distressed by being persecuted*’ (*a* = 4.45, s.e. = 0.10), followed by ‘*I was sure someone wanted to hurt me*’ (*a* = 4.25, s.e. = 0.10). The difficulty parameters for a response of 0–1 (*b*^1^) identified the same four items from the original measure as the most indicative of heightened severity at low levels of endorsement (>0.75 s.d. above average). This included ‘*I was convinced there was a conspiracy against me*’ (*b*^1^ = 0.79, s.e. = 0.02), ‘*I was sure someone wanted to hurt me*’ (*b*^1^ = 0.78, s.e. = 0.01), ‘*I was distressed by being persecuted*’ (*b*^1^ = 0.77, s.e. = 0.01), and ‘*People wanted me to feel threatened, so they stared at me*’ (*b*^1^ = 0.75, s.e. = 0.02). Similarly, for all 10 items, full endorsement (*b*^4^: response 3–4) indicated a severe level of persecutory ideation (>1.50 s.d. above average).

The revised persecution scale retained excellent reliability across the spectrum of paranoia severity, with equivalent *α* values above 0.90 between 0.12 s.d. below and 2.38 s.d. above average levels of paranoia and values above 0.95 between 0.23 and 2.10 s.d. above average. Similar to the original scale, the revised persecution scale demonstrated the highest reliability at elevated levels of paranoia, with a maximum *α* of 0.97 (TI = 39.1, s.e. = 0.16) at 1.21 s.d. above average (see Fig. 1).

#### Score ranges

The total score from the revised 10-item persecution scale has increased precision compared to the original 16-item scale, with a correlation with the participant *θ* scores of *r* = 0.92. As with the original GPTS, the majority of people are unlikely to endorse the persecutory items with an expected score of 2.53 (range 0–40) at the average level of trait paranoia (*θ* = 0). The expected score was 7.46 at 0.5 s.d. above average, 16.8 at 1.0 s.d. above average, 27.7 at 1.5 s.d. above average, and 35.7 at 2 s.d. above average (see Fig. 1).

As shown in Table 2, our recommended cut-off for moderately severe levels of persecutory ideation on the revised persecution scale was 11 (>0.80 s.d. above average). ROC analysis identified 11 as the optimal cut-off (sensitivity = 0.928, 95% CI 0.900–0.953; specificity = 0.852, 95% CI 0.844–0.861) to discriminate patients with a persecutory delusion (*n* = 360) from the non-clinical group (*n* = 7297), with an overall AUC of 0.953 (95% CI 0.943–0.963).

The recommended cut-off for severe persecutory ideation and the likely presence of a persecutory delusion is 18, representing >1.10 s.d. above the average level of paranoia in the population. The ROC analysis demonstrates this cut-off is unlikely to identify incorrectly an individual as having a persecutory delusion when they do not (specificity = 0.93, 95% CI 0.93–0.94), while still correctly identifying the majority of patients with confirmed persecutory delusions (sensitivity = 0.81, 95% CI 0.77–0.85). As shown in Table 3, scores above this level were present in 81% (*n* = 293) of the patients with persecutory delusions (mean = 26.1, s.d. = 9.46).

## Discussion

Empirical research makes it apparent that within the diagnosis of schizophrenia are multiple distinct psychotic experiences, such as paranoia, grandiosity, hallucinations, anhedonia, and thought disorder (e.g. Peralta and Cuesta, 1999; Wigman *et al*., 2011; Peralta *et al*., 2013; Paolini *et al*., 2016). Each of these distinct experiences is on quantitative dimensions in the general population (e.g. Zavos *et al*., 2014; Elahi *et al*., 2017), just as has been found for common emotional disorders (Plomin *et al*., 2009). Precision in the measurement of each psychotic experience is needed. Our particular focus has been on paranoid thinking. Based upon a definition that persecutory ideation concerns unfounded thoughts that others deliberately intend you harm (Freeman and Garety, 2000), the GPTS-Part B scale was developed (Green *et al*., 2008). This was accompanied by the GPTS-Part A, which assesses the related, but less severe phenomena, of ideas of reference. With data from 10 000 people, including over 2000 patients with psychosis, we provide a comprehensive examination of the psychometric properties of the GPTS.

We show that the original GPTS-Persecution scale (Part B) is an adequate assessment of persecutory ideation, with good reliability across the spectrum of paranoia. However, there is a potential for measurement error due to the covariance between several of the items. The original GPTS Reference scale (Part A) stands up less well to the testing. It contains problematic items that are not fully separable from the persecutory ideation scale. We therefore do not recommend this as a stand-alone scale. To overcome these problems, we created a Revised GPTS with stand-alone assessments of persecution ideation and ideas of social reference. Both revised scales have excellent psychometric properties, with high reliability across both non-clinical and clinical levels of paranoia. Importantly, the R-GPTS Persecution scale is most reliable at the severe end of the paranoia spectrum, making it a helpful clinical tool. Future use of the Revised GPTS will produce more precise estimates of the presence of paranoia.

Although we conceive paranoia as having a spectrum of severity in the general population, it is still valuable to ask: what are high and low paranoia levels? When studying paranoia in analogue non-clinical populations, it will be very informative for researchers to specify the level of severity of the phenomenon that is being examined. It will also be beneficial in clinical research to identify the potential presence of persecutory delusions. Our interpretative score ranges will allow this to happen for the first time. Our patient group included several hundred people who were selected for studies on the basis of having a persecutory delusion, enabling precise cut-offs to be identified. Use of the R-GPTS will not only provide more precise estimates of paranoia but will enable better interpretation of the scores.

Where are the weaknesses in the current evaluation? Although our extensive sample includes participants likely representing the full spectrum of paranoia severity, it is important to note that our general population sample was not an epidemiologically representative cohort, which could skew the severity ranges. There is clearly scope for future improvement in the understanding of the total scores for the measure. We also do not report the test-retest reliability of the R-GPTS, although in all likelihood this will remain as high as the original measure. Further, it remains an issue that we cannot know how much of the item endorsement may reflect genuine hostility rather than unfounded paranoia. Nevertheless, we believe the R-GPTS will provide further stimulus for the successful study of paranoia.

## Supplementary material

For supplementary material accompanying this paper visit https://doi.org/10.1017/S0033291719003155. click here to view supplementary material

## Appendix: The Revised Green *et al*., Paranoid Thoughts Scale (R-GPTS)

Please read each of the statements carefully.

They refer to thoughts and feelings you may have had about others over the last month.

**Table:**

	Not at all				Totally
*Part A*					
1. I spent time thinking about friends gossiping about me.	0	1	2	3	4
2. I often heard people referring to me.	0	1	2	3	4
3. I have been upset by friends and colleagues judging me critically.	0	1	2	3	4
4. People definitely laughed at me behind my back.	0	1	2	3	4
5. I have been thinking a lot about people avoiding me.	0	1	2	3	4
6. People have been dropping hints for me.	0	1	2	3	4
7. I believed that certain people were not what they seemed.	0	1	2	3	4
8. People talking about me behind my back upset me.	0	1	2	3	4
*Part B*					
1. Certain individuals have had it in for me.	0	1	2	3	4
2. People wanted me to feel threatened, so they stared at me.	0	1	2	3	4
3. I was certain people did things in order to annoy me.	0	1	2	3	4
4. I was convinced there was a conspiracy against me.	0	1	2	3	4
5. I was sure someone wanted to hurt me.	0	1	2	3	4
6. I couldn't stop thinking about people wanting to confuse me.	0	1	2	3	4
7. I was distressed by being persecuted.	0	1	2	3	4
8. It was difficult to stop thinking about people wanting to make me feel bad.	0	1	2	3	4
9. People have been hostile towards me on purpose.	0	1	2	3	4
10. I was angry that someone wanted to hurt me.	0	1	2	3	4

Think about the last month and indicate the extent of these feelings from 0 (Not at all) to 4 (Totally).

(N.B. Please do not rate items according to any experiences you may have had under the influence of drugs.)
