# Supplementary material for: The revised Green et al., Paranoid Thoughts Scale (R-GPTS): psychometric properties, severity ranges, and clinical cut-offs
Source: Psychol Med. 2019 Nov 20;51(2):244–53. doi: 10.1017/S0033291719003155 (PMC7893506; doi:10.1017/S0033291719003155)
Supplement: Supplementary file 1 [file S0033291719003155sup001.docx]

**SUPPLEMENTARY MATERIALS**

**Table 1.** Exploratory factor analysis with original 32-item GPTS.

|  | **Factor 1** | **Factor 2** |
| --- | --- | --- |
| **GPTS Part A - Reference** |  |  |
| 1. I spent time thinking about friends gossiping about me. |  | 0.843 |
| 1. I often heard people referring to me. |  | 0.623 |
| 1. I have been upset by friends and colleagues judging me critically. |  | 0.845 |
| 1. People definitely laughed at me behind my back. |  | 0.738 |
| 1. I have been thinking a lot about people avoiding me. |  | 0.685 |
| 1. People have been dropping hints for me. |  | 0.585 |
| 1. I believed that certain people were not what they seemed. |  | 0.619 |
| 1. People talking about me behind my back upset me. |  | 0.829 |
| 1. I was convinced that people were singling me out. | 0.449 | 0.454 |
| 1. I was certain that people have followed me. | 0.633 |  |
| 1. Certain people were hostile towards me personally. | 0.534 |  |
| 1. People have been checking up on me. | 0.479 | 0.347 |
| 1. I was stressed out by people watching me. | 0.521 | 0.367 |
| 1. I was frustrated by people laughing at me. |  | 0.605 |
| 1. I was worried by people’s undue interest in me. | 0.464 | 0.395 |
| 1. It was hard to stop thinking about people talking about me behind my back. |  | 0.638 |
| **Part B – Persecution** |  |  |
| 1. Certain individuals have had it in for me | 0.798 |  |
| 1. I have definitely been persecuted. | 0.899 |  |
| 1. People have intended me harm | 1.008 |  |
| 1. People wanted me to feel threatened, so they stared at me | 0.626 |  |
| 1. I was certain people did things in order to annoy me. | 0.593 |  |
| 1. I was convinced there was a conspiracy against me | 0.84 |  |
| 1. I was sure someone wanted to hurt me | 1.033 |  |
| 1. I was distressed by people wanting to harm me in some way | 1.003 |  |
| 1. I was preoccupied with thoughts of people trying to upset me deliberately | 0.802 |  |
| 1. I couldn’t stop thinking about people wanting to confuse me. | 0.663 |  |
| 1. I was distressed by being persecuted | 0.936 |  |
| 1. I was annoyed because others wanted to deliberately upset me | 0.727 |  |
| 1. The thought that people were persecuting me played on my mind | 0.927 |  |
| 1. It was difficult to stop thinking about people wanting to make me feel bad | 0.796 |  |
| 1. People have been hostile towards me on purpose | 0.815 |  |
| 1. I was angry that someone wanted to hurt me | 0.776 |  |

Table 2. Item response theory parameters for the original GPTS Persecution scale. Standard errors are shown in parentheses.

| **Item** | | ***a*** | ***b1*** | ***b2*** | ***b3*** | ***b4*** |
| --- | --- | --- | --- | --- | --- | --- |
| 1. | Certain individuals have had it in for me | 3.06 (0.06) | 0.22 (0.01) | 0.72 (0.02) | 1.28 (0.02) | 1.70 (0.03) |
| 2. | I have definitely been persecuted. | 3.64 (0.07) | 0.62 (0.01) | 1.00 (0.02) | 1.43 (0.02) | 1.79 (0.03) |
| 3. | People have intended me harm | 3.98 (0.09) | 0.73 (0.01) | 1.09 (0.02) | 1.44 (0.02) | 1.80 (0.03) |
| 4. | People wanted me to feel threatened, so they stared at me | 2.84 (0.06) | 0.76 (0.02) | 1.16 (0.02) | 1.60 (0.03) | 2.05 (0.03) |
| 5. | I was certain people did things in order to annoy me. | 2.45 (0.04) | -0.09 (0.02) | 0.47 (0.02) | 1.09 ((0.02) | 1.62 (0.03) |
| 6. | I was convinced there was a conspiracy against me | 3.76 (0.08) | 0.79 (0.02) | 1.08 (0.02) | 1.42 (0.02) | 1.76 (0.03) |
| 7. | I was sure someone wanted to hurt me | 4.65 (0.10) | 0.78 (0.01) | 1.09 (0.02) | 1.38 (0.02) | 1.69 (0.02) |
| 8. | I was distressed by people wanting to harm me in some way | 4.82 (0.11) | 0.72 (0.01) | 1.04 (0.02) | 1.37 (0.02) | 1.73 (0.02) |
| 9. | I was preoccupied with thoughts of people trying to upset me deliberately | 4.15 (0.08) | 0.38 (0.01) | 0.78 (0.02) | 1.19 (0.02) | 1.63 (0.02) |
| 10. | I couldn’t stop thinking about people wanting to confuse me. | 3.16 (0.06) | 0.65 (0.02) | 1.03 (0.02) | 1.49 (0.02) | 1.90 (0.03) |
| 11. | I was distressed by being persecuted | 5.37 (0.12) | 0.76 (0.01) | 1.05 (0.02) | 1.38 (0.02) | 1.74 (0.02) |
| 12. | I was annoyed because others wanted to deliberately upset me | 4.15 (0.08) | 0.41(0.01) | 0.81 (0.02) | 1.23 (0.02) | 1.63 (0.02) |
| 13. | The thought that people were persecuting me played on my mind | 5.07 (0.11) | 0.66 (0.01) | 0.95 (0.02) | 1.29 (0.02) | 1.66 (0.02) |
| 14. | It was difficult to stop thinking about people wanting to make me feel bad | 4.25 (0.08) | 0.41 (0.01) | 0.79 (0.02) | 1.19 (0.02) | 1.58 (0.02) |
| 15. | People have been hostile towards me on purpose | 3.45 (0.07) | 0.37 (0.01) | 0.81 (0.02) | 1.25 (0.02) | 1.67 (0.02) |
| 16. | I was angry that someone wanted to hurt me | 3.72 (0.08) | 0.63 (0.01) | 0.98 (0.02) | 1.34 (0.02) | 1.69 (0.03) |

Note: *a* = discrimination, *b* = difficulty parameters at the category thresholds between 0-1 (*b_1_*), 1-2 (*b_2_*), 2-3 (*b_3_*) and 3-4 (*b_4_*)

**Figure 1**. Test information (TI) with standard errors and expected score across the theta distribution


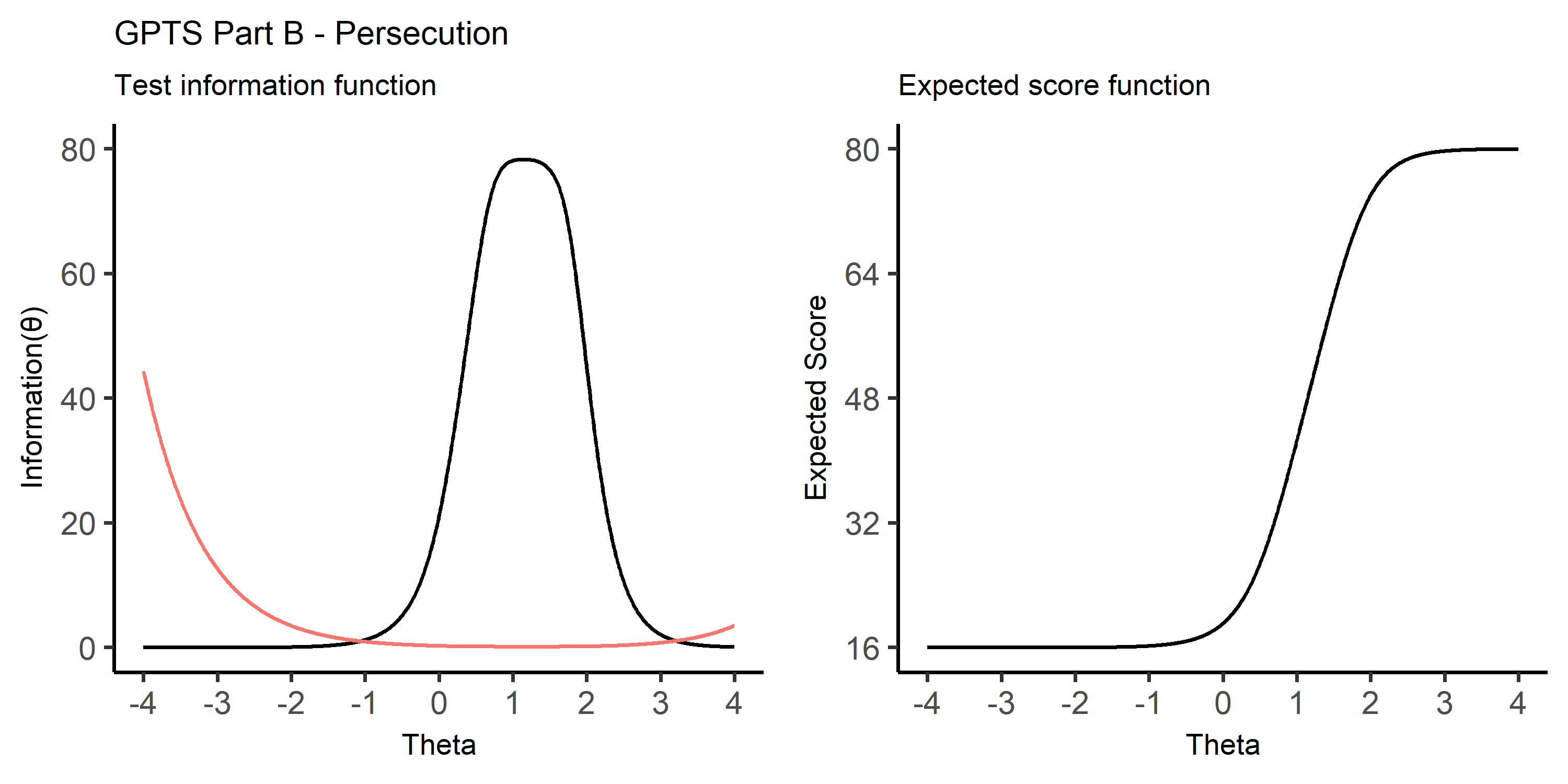


**Table 3**. EFA factor loadings for the Revised GPTS

|  | **Factor 1** | **Factor 2** |
| --- | --- | --- |
| **GPTS Part A - Reference** |  |  |
| 1. I spent time thinking about friends gossiping about me. |  | 0.843 |
| 1. I often heard people referring to me. |  | 0.623 |
| 1. I have been upset by friends and colleagues judging me critically. |  | 0.845 |
| 1. People definitely laughed at me behind my back. |  | 0.738 |
| 1. I have been thinking a lot about people avoiding me. |  | 0.685 |
| 1. People have been dropping hints for me. |  | 0.585 |
| 1. I believed that certain people were not what they seemed. |  | 0.619 |
| 1. People talking about me behind my back upset me. |  | 0.829 |
| **Part B – Persecution** |  |  |
| 1. Certain individuals have had it in for me | 0.798 |  |
| 1. People wanted me to feel threatened, so they stared at me | 0.626 |  |
| 1. I was certain people did things in order to annoy me. | 0.593 |  |
| 1. I was convinced there was a conspiracy against me | 0.840 |  |
| 1. I was sure someone wanted to hurt me | 1.033 |  |
| 1. I couldn’t stop thinking about people wanting to confuse me. | 0.663 |  |
| 1. I was distressed by being persecuted | 0.936 |  |
| 1. It was difficult to stop thinking about people wanting to make me feel bad | 0.796 |  |
| 1. People have been hostile towards me on purpose | 0.815 |  |
| 1. I was angry that someone wanted to hurt me | 0.776 |  |
